# Supplementary material for: Synthetic data analysis for early detection of Alzheimer progression through machine learning algorithms
Source: PeerJ Comput Sci. 2024 Dec 12;10:e2437. doi: 10.7717/peerj-cs.2437 (PMC11784714; doi:10.7717/peerj-cs.2437)
Supplement: Supplemental Information 2 [file peerj-cs-10-2437-s002.pdf]

SYNTHETIC DATA ANALYSIS FOR EARLY DETECTION OF ALZHEIMER PROGRESSION THROUGH MACHINE LEARNING ALGORITHMS

Ana G. Sánchez-Reyna, Ricardo Mendoza-González, Huizilopoztli Luna-García, José M. Celaya-Padilla, Jorge A. Morgan-Benita, Carlos H. Espino-Salinas, Jorge I. Galván-Tejada, David Rondon and Klinge O. Villalba-Condori

Supplemental Experimental Results

As part of the work developed, the following extra results were obtained.

These features were processed by RFE and AIC, obtaining diverse sets of features that were then integrated into the ML models within the ensemble (see Table S1 and Table S2).

Experiment	Dataset	Features
CNvSMC	Orig	“FAQ”
(phase 1)	Syn	“AGE”, “ADAS11”, “ADAS13” and “TRABSCOR”
	Custom	“AGE” and “ADAS13”
CNvsEMCI	Orig	“PTGENDER”, “CDRSB” and “FAQ”
(phase 1)	Syn	“PTGENDER”, “CDRSB” and “FAQ”
	Custom	“PTGENDER”, “CDRSB” and “FAQ”
CNvsLMCI	Orig	“CDRSB”, “LDELTOTAL” and “FAQ”
(phase 1)	Syn	“CDRSB”, “LDELTOTAL” and “FAQ”
	Custom	“CDRSB”, “LDELTOTAL” and “FAQ”
CNvsAD	Orig	“FAQ”
(phase 1)	Syn	“FAQ”
	Custom	“FAQ”
SMCvsEMCI	Orig	“AGE”, “CDRSB” and “TRABSCOR”
(phase 1)	Syn	“AGE”, “CDRSB” and “TRABSCOR”
	Custom	“AGE”, “CDRSB” and “TRABSCOR”
SMCvsLMCI	Orig	“AGE”, “CDRSB”, “ADAS11”, “ADAS13”, “RAVLT.immediate”, “LDELTOTAL” and “TRABSCOR”
(phase 1)	Syn	“PTGENDER”, “PTMARRY”, “CDRSB”, “ADAS11”, “ADAS13”, “ADASQ4”, “MMSE” and “LDELTOTAL”
	Custom	“ADAS13” and “LDELTOTAL”
SMCvsAD	Orig	“CDRSB”, “ADAS13”, “ADASQ4”, “RAVLT.immediate”, “RAVLT.learning”, “LDELTOTAL” and “FAQ”
(phase 1)	Syn	“CDRSB”, “ADAS13”, “ADASQ4”, “RAVLT.immediate”, “LDELTOTAL” and “FAQ”
	Custom	“ADAS13”
EMCIvsLMCI	Orig	“AGE”, “ADAS11”, “ADAS13”, “RAVLT.immediate”, “LDELTOTAL”, “TRABSCOR” and “FAQ”
(phase 1)	Syn	“AGE”, “ADAS11”, “ADAS13”, “ADASQ4”, “MMSE”, “RAVLT.immediate”, “LDELTOTAL”, “TRABSCOR” and “FAQ”
	Custom	“AGE”, “ADAS13” and “RAVLT.immediate”
EMCIvsAD	Orig	“CDRSB”, “ADAS13”, “LDELTOTAL”, “TRABSCOR” and “FAQ”
(phase 1)	Syn	“CDRSB”, “ADAS11”, “ADAS13”, “RAVLT.immediate”, “RAVLT.learning”, “LDELTOTAL” and “TRABSCOR”
	Custom	“CDRSB” and “ADAS13”
LMCIvsAD	Orig	“RAVLT.learning”
(phase 1)	Syn	“AGE”, “CDRSB”, “RAVLT.immediate”, “RAVLT.learning” and “FAQ”
	Custom	“AGE” and “CDRSB”

Table S1. Summary of the features of each dataset from phase 1 of the experiments.

The Table S3 shows the comparison between the number of subjects in the original data set according to the degree of cognitive impairment with the number of subjects in the synthetic data set.

The mean and standard deviation were calculated to analyze the central tendency and statistical dispersion of the data, for the CN vs SMC (phase 1) datasets, the results of these statistical calculations

Experiment	Dataset	Features
CNvsMCI (phase 2)	Orig	“PTGENDER”, “CDRSB” and “ADAS11”
	Syn	“PTGENDER” and “CDRSB”
	Custom	“CDRSB”
CNvsAD (phase 2)	Orig	“CDRSB”, “ADASQ4”, “RAVLT.learning”
	Syn	“RAVLT.immediate”, “FAQ”
	Custom	“RAVLT.immediate”, “FAQ”
MCIvsAD (phase 2)	Orig	“FAQ”
	Syn	“CDRSB”, “ADAS13”, “RAVLT.immediate”, “RAVLT.learning”, “LDELTOTAL” and “FAQ”
	Custom	“ADAS13”

Table S2. Summary of the features of each dataset from phase 2 of the experiments.

Dataset	Orig Dataset No. Subjects		Syn Dataset No. Subjects	
CNvsSMC (phase 1)	CN	SMC	CN	SMC
	85	49	85	85
CNvsEMCI (phase 1)	CN	EMCI	CN	EMCI
	85	88	88	88
CNvsLMCI (phase 1)	CN	LMCI	CN	LMCI
	85	79	85	85
CNvsAD (phase 1)	CN	AD	CN	AD
	85	22	85	85
SMCvsEMCI (phase 1)	SMC	EMCI	SMC	EMCI
	49	88	88	88
SMCvsLMCI (phase 1)	SMC	LMCI	SMC	LMCI
	49	79	79	79
SMCvsAD (phase 1)	SMC	AD	SMC	AD
	49	22	49	49
EMCIvsLMCI (phase 1)	EMCI	LMCI	EMCI	LMCI
	88	79	88	88
EMCIvsAD (phase 1)	EMCI	AD	EMCI	AD
	88	22	88	88
LMCIvsAD (phase 1)	LMCI	AD	LMCI	AD
	79	22	79	79
CNvsMCI (phase 2)	CN	MCI	CN	MCI
	134	167	167	167
CNvsAD (phase 2)	CN	AD	CN	AD
	134	22	134	134
MCIvsAD (phase 2)	CN	AD	MCI	AD
	167	22	167	167

Table S3. Comparison of the number of subjects in the original dataset and the synthetic dataset, according to the different experimental datasets.

are presented in Table S4.

The mean and standard deviation were calculated to analyze the central tendency and statistical dispersion of the data, for the CN vs EMCI (phase 1), the results of these statistical calculations are presented in Table S5.

The mean and standard deviation were calculated to analyze the central tendency and statistical dispersion of the data, for the CN vs LMCI (phase 1) datasets, the results of these statistical calculations are presented in Table S6.

The mean and standard deviation were calculated to analyze the central tendency and statistical

Feature	Mean Orig	SD Orig	Mean Syn	SD Syn
DX.bl	0.3657	0.4834	0.5000	0.5015
AGE	69.0948	5.9821	68.3653	5.9159
PTGENDER	0.2388	0.4280	0.2176	0.4139
PTMARRY	1.3731	0.6566	1.3647	0.6499
CDRSB	0.1381	0.4309	0.1441	0.4533
ADAS11	5.7537	2.9483	5.5591	2.8642
ADAS13	8.3433	3.9413	8.1708	3.8903
ADASQ4	2.3582	1.5139	2.3471	1.4966
MMSE	28.8507	1.3683	28.9588	1.2842
RAVLT.immediate	45.0821	10.1010	45.8000	11.0794
RAVLT.learning	5.9776	2.1642	6.0353	2.1481
RAVLT.forgetting	3.7239	3.1940	3.6706	3.2450
LDELTOTAL	12.8358	3.5798	12.8588	3.6460
TRABSCOR	76.9552	28.7760	76.9706	29.0163
FAQ	0.1791	0.8571	0.2059	0.9095

Table S4. Cognitive Normal vs Subjective Memory Concern (phase 1) datasets. Mean and standard deviation of all the features used in these models, according to the dataset to which they belong, Orig is the Original dataset and Syn is the Synthetic dataset.

Feature	Mean Orig	SD Orig	Mean Syn	SD Syn
DX.bl	0.5087	0.5014	0.5000	0.5014
AGE	70.5671	7.0481	70.6591	7.0496
PTGENDER	0.3353	0.4735	0.3409	0.4754
PTMARRY	1.3642	0.7395	1.3580	0.7346
CDRSB	0.6676	1.1145	0.6563	1.1083
ADAS11	6.3679	3.7740	6.3616	3.7479
ADAS13	9.4661	5.5559	9.4241	5.5266
ADASQ4	2.8324	2.1297	2.7955	2.1309
MMSE	28.3468	2.0954	28.3409	2.0778
RAVLT.immediate	43.3121	11.4904	43.2614	11.4213
RAVLT.learning	5.6821	2.5852	5.6818	2.5637
RAVLT.forgetting	3.4798	3.3646	3.5284	3.3669
LDELTOTAL	12.2948	4.4014	12.3011	4.3694
TRABSCOR	90.6647	40.3040	90.5852	40.0783
FAQ	1.2775	3.1519	1.2557	3.1291

Table S5. Cognitive Normal vs Early Mild Cognitive Impairment (phase 1) datasets. Mean and standard deviation of all the features used in these models, according to the dataset to which they belong, Orig is the Original dataset and Syn is the Synthetic dataset.

dispersion of the data, for the CN vs AD (phase 1) datasets, the results of these statistical calculations are presented in Table S7.

The mean and standard deviation were calculated to analyze the central tendency and statistical dispersion of the data, for the SMC vs EMCI (phase 1) datasets, the results of these statistical calculations are presented in Table S8.

The mean and standard deviation were calculated to analyze the central tendency and statistical dispersion of the data, for the SMC vs LMCI (phase 1) datasets, the results of these statistical calculations are presented in Table S9.

The mean and standard deviation were calculated to analyze the central tendency and statistical dispersion of the data, for the SMC vs AD (phase 1) datasets, the results of these statistical calculations are presented in Table S10.

The mean and standard deviation were calculated to analyze the central tendency and statistical dispersion of the data, for the EMCI vs LMCI (phase 1) datasets, the results of these statistical calculations

Feature	Mean Orig	SD Orig	Mean Syn	SD Syn
DX.bl	0.4817	0.5012	0.5000	0.5015
AGE	71.1726	6.6312	71.3559	6.5879
PTGENDER	0.3171	0.4668	0.3118	0.4646
PTMARRY	1.4756	0.7630	1.5000	0.7712
CDRSB	1.2622	1.9950	1.4000	2.1951
ADAS11	9.5995	7.0982	9.9979	7.4820
ADAS13	14.5873	10.5089	15.1568	10.9968
ADASQ4	4.2317	3.0589	4.3647	3.1297
MMSE	27.1159	3.5000	26.9176	3.8252
RAVLT.immediate	36.2927	12.7578	35.6588	13.0923
RAVLT.learning	4.7073	2.5525	4.6706	2.5368
RAVLT.forgetting	4.2012	2.7917	4.2471	2.7965
LDELTOTAL	8.9573	5.5665	8.7706	5.6022
TRABSCOR	130.0732	86.0649	133.5529	87.5929
FAQ	3.0549	5.8794	3.4941	6.3797

Table S6. Cognitive Normal vs Late Mild Cognitive Impairment (phase 1) datasets. Mean and standard deviation of all the features used in these models, according to the dataset to which they belong, Orig is the Original dataset and Syn is the Synthetic dataset.

Feature	Mean Orig	SD Orig	Mean Syn	SD Syn
DX.bl	0.2056	0.4060	0.5000	0.5015
AGE	71.3318	7.0128	72.8959	7.5961
PTGENDER	0.3271	0.4714	0.4588	0.4998
PTMARRY	1.3738	0.6662	1.3235	0.6491
CDRSB	1.1916	2.4722	2.6294	3.0636
ADAS11	8.2864	6.8090	12.2781	8.5512
ADAS13	12.1555	9.9124	18.2487	12.2575
ADASQ4	3.4019	2.8413	5.1176	3.3403
MMSE	27.7196	2.7738	26.2000	3.4256
RAVLT.immediate	39.7664	12.9859	33.1294	13.7645
RAVLT.learning	4.9626	2.7675	3.6412	2.8670
RAVLT.forgetting	3.7944	2.8409	3.9412	2.3602
LDELTOTAL	10.7944	5.7326	7.3176	6.6318
TRABSCOR	110.2523	81.0888	154.3706	101.7516
FAQ	3.5607	8.0554	8.1353	10.3968

Table S7. Cognitive Normal vs Alzheimer Disease (phase 1) datasets. Mean and standard deviation of all the features used in these models, according to the dataset to which they belong, Orig is the Original dataset and Syn is the Synthetic dataset.

are presented in Table S11.

The mean and standard deviation were calculated to analyze the central tendency and statistical dispersion of the data, for the EMCI vs AD (phase 1) datasets, the results of these statistical calculations are presented in Table S12.

The mean and standard deviation were calculated to analyze the central tendency and statistical dispersion of the data, for the LMCI vs AD (phase 1) datasets, the results of these statistical calculations are presented in Table S13.

The mean and standard deviation were calculated to analyze the central tendency and statistical dispersion of the data, for the CN vs MCI (phase 1) datasets, the results of these statistical calculations are presented in Table S14.

The mean and standard deviation were calculated to analyze the central tendency and statistical dispersion of the data, for the CN vs AD (phase 2) datasets, the results of these statistical calculations are presented in Table S15.

Feature	Mean Orig	SD Orig	Mean Syn	SD Syn
DX.bl	0.6423	0.4811	0.5000	0.5014
AGE	69.4423	7.1491	68.6222	6.8422
PTGENDER	0.3796	0.4871	0.3239	0.4693
PTMARRY	1.3285	0.7387	1.3239	0.7113
CDRSB	0.8978	1.2159	0.7358	1.1401
ADAS11	6.8955	4.0555	6.4491	3.8311
ADAS13	10.4210	5.8478	9.7844	5.5436
ADASQ4	3.1679	2.1948	2.9659	2.0809
MMSE	28.2628	2.2072	28.4943	2.0284
RAVLT.immediate	43.4453	12.4681	44.4659	12.8223
RAVLT.learning	5.7226	2.5945	5.8466	2.4875
RAVLT.forgetting	3.4234	3.4805	3.4148	3.4432
LDELTOTAL	11.7007	4.7129	11.9659	4.5505
TRABSCOR	96.1533	43.4770	91.9943	41.5573
FAQ	1.7591	3.4990	1.4318	3.1832

Table S8. Subjective Memory Concern vs Early Mild Cognitive Impairment (phase 1) datasets. Mean and standard deviation of all the features used in these models, according to the dataset to which they belong, Orig is the Original dataset and Syn is the Synthetic dataset.

Feature	Mean Orig	SD Orig	Mean Syn	SD Syn
DX.bl	0.6172	0.4880	0.5000	0.5016
AGE	70.1391	6.7487	69.3905	6.5858
PTGENDER	0.3594	0.4817	0.3165	0.4666
PTMARRY	1.4688	0.7729	1.4430	0.7529
CDRSB	1.6758	2.1209	1.3956	2.0096
ADAS11	11.0730	7.3798	9.8991	7.1551
ADAS13	17.0496	10.6510	15.2535	10.4112
ADASQ4	4.9844	3.0013	4.4684	2.9748
MMSE	26.6797	3.7625	27.1835	3.5599
RAVLT.immediate	34.4609	13.4626	37.3038	14.6408
RAVLT.learning	4.4766	2.5066	4.8228	2.5251
RAVLT.forgetting	4.3438	2.7506	4.2215	2.9575
LDELTOTAL	7.3828	5.2693	8.3987	5.4655
TRABSCOR	147.0313	90.3705	134.3165	86.4932
FAQ	4.0703	6.3633	3.3671	5.9278

Table S9. Subjective Memory Concern vs Late Mild Cognitive Impairment (phase 1) datasets. Mean and standard deviation of all the features used in these models, according to the dataset to which they belong, Orig is the Original dataset and Syn is the Synthetic dataset.

The mean and standard deviation were calculated to analyze the central tendency and statistical dispersion of the data, for the MCI vs AD (phase 2) datasets, the results of these statistical calculations are presented in Table S16.

Feature	Mean Orig	SD Orig	Mean Syn	SD Syn
DX.bl	0.3099	0.4657	0.5000	0.5026
AGE	69.5493	7.3505	71.3765	8.4053
PTGENDER	0.4085	0.4950	0.4796	0.5022
PTMARRY	1.3099	0.6232	1.2653	0.6016
CDRSB	1.9014	2.8380	2.8112	3.1084
ADAS11	10.2772	7.6048	12.4253	8.0480
ADAS13	15.3617	10.8151	18.7212	11.3695
ADASQ4	4.3380	2.9758	5.3469	3.1298
MMSE	27.2394	3.1143	26.3367	3.3211
RAVLT.immediate	38.2254	15.0325	33.8776	14.7880
RAVLT.learning	4.6761	2.8323	3.7755	2.8701
RAVLT.forgetting	3.8451	2.8216	3.9796	2.4662
LDELTOTAL	8.8873	6.1378	6.8469	6.3560
TRABSCOR	130.7746	93.0148	155.1531	100.1339
FAQ	5.6479	9.3107	8.3980	10.5251

Table S10. Subjective Memory Concern vs Alzheimer Disease (phase 1) datasets. Mean and standard deviation of all the features used in these models, according to the dataset to which they belong, Orig is the Original dataset and Syn is the Synthetic dataset.

Feature	Mean Orig	SD Orig	Mean Syn	SD Syn
DX.bl	0.4731	0.5008	0.5000	0.5014
AGE	71.4204	7.4366	71.5716	7.3282
PTGENDER	0.4311	0.4967	0.4261	0.4959
PTMARRY	1.4371	0.8254	1.4659	0.8343
CDRSB	1.8653	1.9220	1.9773	2.0770
ADAS11	10.4670	7.0230	10.8901	7.3314
ADAS13	16.1796	10.2134	16.7594	10.5791
ADASQ4	4.8623	2.9998	5.0000	3.0388
MMSE	26.6647	3.6016	26.4773	3.8663
RAVLT.immediate	35.1078	13.5602	34.4375	13.7157
RAVLT.learning	4.5210	2.7700	4.4659	2.7379
RAVLT.forgetting	3.9461	3.1070	3.9886	3.0799
LDELTOTAL	8.0958	5.6283	7.9034	5.6261
TRABSCOR	144.8683	81.3034	146.7898	82.1794
FAQ	4.2994	6.0445	4.6705	6.3941

Table S11. Early Mild Cognitive Impairment vs Late Mild Cognitive Impairment (phase 1) datasets. Mean and standard deviation of all the features used in these models, according to the dataset to which they belong, Orig is the Original dataset and Syn is the Synthetic dataset.

Feature	Mean Orig	SD Orig	Mean Syn	SD Syn
DX.bl	0.2000	0.4018	0.5000	0.5014
AGE	71.7036	8.1318	73.2920	8.3177
PTGENDER	0.5000	0.5023	0.5739	0.4959
PTMARRY	1.3182	0.7654	1.2841	0.7083
CDRSB	2.1091	2.3358	3.2472	2.7589
ADAS11	9.6393	6.9066	13.2857	8.3230
ADAS13	14.6393	9.9475	19.9960	11.6304
ADASQ4	4.3818	2.9707	5.7557	3.1172
MMSE	27.0182	3.0948	25.7216	3.4237
RAVLT.immediate	37.8727	14.5641	31.8182	14.2083
RAVLT.learning	4.6727	3.0833	3.4318	2.9887
RAVLT.forgetting	3.4182	3.2464	3.6989	2.6759
LDELTOTAL	9.4364	6.1711	6.4148	6.4554
TRABSCOR	133.2545	78.0732	169.4205	94.3624
FAQ	5.4364	7.9972	9.5000	9.9794

Table S12. Early Mild Cognitive Impairment vs Alzheimer Disease (phase 1) datasets. Mean and standard deviation of all the features used in these models, according to the dataset to which they belong, Orig is the Original dataset and Syn is the Synthetic dataset.

Feature	Mean Orig	SD Orig	Mean Syn	SD Syn
DX.bl	0.2178	0.4148	0.5000	0.5016
AGE	72.7881	7.4398	73.7709	7.7463
PTGENDER	0.4851	0.5023	0.5570	0.4983
PTMARRY	1.4950	0.8078	1.3924	0.7473
CDRSB	3.2030	2.5828	3.8861	2.6786
ADAS11	15.1781	7.7539	16.4808	7.5890
ADAS13	23.4157	10.5209	25.2150	10.0444
ADASQ4	6.7921	2.6620	7.2405	2.4323
MMSE	24.9010	3.9103	24.4494	3.6180
RAVLT.immediate	25.9901	8.9280	24.5063	7.9302
RAVLT.learning	3.0000	2.2136	2.4367	2.0204
RAVLT.forgetting	4.5842	2.0845	4.4557	1.8009
LDELTOTAL	3.7624	3.5246	2.9304	3.4698
TRABSCOR	201.0396	90.3411	211.5949	90.9935
FAQ	8.6931	8.8111	11.1013	9.5891

Table S13. Late Mild Cognitive Impairment vs Alzheimer Disease (phase 1) datasets. Mean and standard deviation of all the features used in these models, according to the dataset to which they belong, Orig is the Original dataset and Syn is the Synthetic dataset.

Feature	Mean Orig	SD Orig	Mean Syn	SD Syn
DX.bl	0.5548	0.4978	0.5000	0.5008
AGE	70.3850	6.9142	70.2542	6.7219
PTGENDER	0.3455	0.4763	0.3383	0.4738
PTMARRY	1.4086	0.7544	1.4132	0.7493
CDRSB	1.0963	1.6928	0.9910	1.6385
ADAS11	8.3687	6.0540	8.1796	5.8603
ADAS13	12.6910	8.9345	12.3683	8.6373
ADASQ4	3.7475	2.7476	3.6377	2.6819
MMSE	27.6379	3.0318	27.7844	2.9216
RAVLT.immediate	39.5482	13.1010	40.1287	12.9094
RAVLT.learning	5.1694	2.6168	5.3473	2.5934
RAVLT.forgetting	3.8472	3.1427	3.9132	3.1559
LDELTOTAL	10.2060	5.3645	10.4850	5.2343
TRABSCOR	114.6346	71.8871	110.2844	69.8493
FAQ	2.4651	4.9749	2.2216	4.7791

Table S14. Cognitive Normal vs Mild Cognitive Impairment (phase 2) datasets. Mean and standard deviation of all the features used in these models, according to the dataset to which they belong, Orig is the Original dataset and Syn is the Synthetic dataset.

Feature	Mean Orig	SD Orig	Mean Syn	SD Syn
DX.bl	0.1410	0.3492	0.5000	0.5009
AGE	69.9654	6.7366	72.0743	7.5879
PTGENDER	0.3077	0.4630	0.4701	0.5000
PTMARRY	1.3590	0.6520	1.3209	0.6488
CDRSB	0.9006	2.1209	2.6959	3.0313
ADAS11	7.6517	5.9930	12.2051	8.4018
ADAS13	11.2478	8.6302	18.2424	11.9524
ADASQ4	3.1667	2.5217	5.1567	3.2322
MMSE	28.0962	2.4673	26.1866	3.4330
RAVLT.immediate	41.6987	12.7068	33.3507	14.2012
RAVLT.learning	5.3333	2.6011	3.6978	2.8630
RAVLT.forgetting	3.7756	2.9894	3.9254	2.3832
LDELTOTAL	11.2372	5.2611	7.2127	6.5064
TRABSCOR	100.6218	70.9902	156.6791	100.6630
FAQ	2.5833	6.8598	8.0560	10.2931

Table S15. Cognitive Normal vs Alzheimer Disease (phase 2) datasets. Mean and standard deviation of all the features used in these models, according to the dataset to which they belong, Orig is the Original dataset and Syn is the Synthetic dataset.

Feature	Mean Orig	SD Orig	Mean Syn	SD Syn
DX.bl	0.1164	0.3216	0.5000	0.5008
AGE	71.8683	7.6571	73.1895	7.8143
PTGENDER	0.4656	0.5001	0.5838	0.4937
PTMARRY	1.4180	0.8055	1.3503	0.7311
CDRSB	2.2937	2.3022	3.6123	2.7232
ADAS11	11.4849	7.5309	14.7693	8.2621
ADAS13	17.6648	10.8037	22.4190	11.3379
ADASQ4	5.2381	3.0565	6.4581	2.8992
MMSE	26.2963	3.6477	25.0449	3.5676
RAVLT.immediate	33.4762	13.6294	28.0719	12.4276
RAVLT.learning	4.1587	2.8183	2.9760	2.6291
RAVLT.forgetting	3.9630	2.9434	4.0150	2.3191
LDELTOTAL	7.3280	5.7712	4.7904	5.5387
TRABSCOR	156.4974	87.2183	192.8802	94.0123
FAQ	5.8042	7.6554	10.2934	9.7758

Table S16. Mild Cognitive Impairment vs Alzheimer Disease (phase 2) datasets. Mean and standard deviation of all the features used in these models, according to the dataset to which they belong, Orig is the Original dataset and Syn is the Synthetic dataset.
